# Emotional over- and under-eating in early childhood are learned not inherited

**DOI:** 10.1038/s41598-017-09519-0

**Published:** 2017-08-22

**Authors:** Moritz Herle, Alison Fildes, Silje Steinsbekk, Fruhling Rijsdijk, Clare H. Llewellyn

**Affiliations:** 1 Department of Behavioural Science and Health, University College London, London, United Kingdom; 2 School of Psychology, University of Leeds, Leeds, United Kingdom; 3 Department of Psychology, Norwegian University of Science and Technology, Trondheim, Norway; 4 Social, Genetic and Developmental Psychiatry Centre, Institute of Psychology, Psychiatry and Neuroscience, King’s College London, London, UK

## Abstract

Emotional overeating (EOE) has been associated with increased obesity risk, while emotional undereating (EUE) may be protective. Interestingly, EOE and EUE tend to correlate positively, but it is unclear whether they reflect different aspects of the same underlying trait, or are distinct behaviours with different aetiologies. Data were from 2054 five-year-old children from the Gemini twin birth cohort, including parental ratings of child EOE and EUE using the Child Eating Behaviour Questionnaire. Genetic and environmental influences on variation and covariation in EUE and EOE were established using a bivariate Twin Model. Variation in both behaviours was largely explained by aspects of the environment completely shared by twin pairs (EOE: C = 90%, 95% CI: 89%-92%; EUE: C = 91%, 95% CI: 90%-92%). Genetic influence was low (EOE: A = 7%, 95% CI: 6%-9%; EUE: A = 7%, 95% CI: 6%-9%). EOE and EUE correlated positively (r = 0.43, p < 0.001), and this association was explained by common shared environmental influences (BivC = 45%, 95% CI: 40%-50%). Many of the shared environmental influences underlying EUE and EOE were the same (r_C_ = 0.50, 95% CI: 0.44, 0.55). Childhood EOE and EUE are etiologically distinct. The tendency to eat more or less in response to emotion is learned rather than inherited.

## Introduction

Emotional eating is the tendency to change one’s eating behaviour in response to negative emotions^1^. Research with adults has shown that some people tend to consume *more* in stressful situations (so-called emotional *overeating*, [EOE]), whereas others experience a decrease in appetite when distressed and eat *less* (so-called emotional *under-eating*, [EUE])^1, 2^. The tendency to either over- or under-eat in response to negative emotion appears to emerge in the preschool years^3^.

Understanding the aetiologies of these behaviours in early life is important because EOE has been hypothesized to play a causal role in overweight, and EUE in under-weight^4^. There has been some support for these hypotheses insofar as EOE has been associated with higher weight cross-sectionally^5–10^, and with weight gain longitudinally from 5–6 years to 6–8 years^11^ and from 4 to 8 years^12^. On the other hand, EUE has been negatively associated with weight^9, 13, 14^. However, null findings have sometimes been reported in cross-sectional studies for both EOE^15, 16^ and EUE^9, 15^.

Despite their differing associations with weight, EOE and EUE tend to be positively correlated^9, 17, 18^, indicating that some children have an underlying tendency to *both* under- *and* overeat in response to negative emotions. This raises the question as to whether these two behaviours reflect different aspects of the same underlying trait (i.e. a tendency to *both* over- *and* under-eat in response to negative emotion) with a common aetiology, or are distinct traits with different aetiologies. Twin studies offer a powerful method for establishing the extent to which behaviour is shaped by genes and environments; and can also elucidate shared aetiology by quantifying the extent to which different behaviours share common or distinct genetic and environmental influences. We have conducted the only paediatric twin study of EOE, finding this behaviour to be influenced largely by aspects of the environment completely shared by twin pairs, in both toddlerhood and early childhood; with genetic factors playing a minimal role^19^. To date there have been no twin studies of EUE in adults or children. The objectives of this study were to use a twin design to: (i) establish for the first time the relative genetic and environmental contributions to EUE in early childhood, and (ii) establish the extent to which EOE and EUE share a common genetic and environmental aetiology.

## Methods

### Participants

Participants were from Gemini, a population-based birth cohort of 2402 British families with twins born in 2007. Gemini is the largest twin study ever set up to study genetic and environmental influences on early growth, and has a particular focus on the role of early eating behaviour^20^. Between March and December 2007, the Office for National Statistics contacted all families in England and Wales with live twin births (N = 6725) for consent to pass on their contact details to the Gemini research team. 2402 families (n = 4804 individual twins) completed and returned the baseline questionnaire; they constitute the Gemini sample, and include 749 monozygotic pairs [MZs], 1616 dizygotic pairs [DZs], and 37 pairs of unknown zygosity. The Gemini families are representative of UK twins when compared on zygosity, sex, gestational age and birth weight. As with most cohort studies, compared to national UK statistics Gemini parents have below average BMIs and healthier eating habits, and white and married couples are overrepresented in the sample. The current study includes data on EOE and EUE collected when the twins were approximately five years old (n = 2054 individual children; mean, 5.15 years; SD, 0.13). Ethical approval was granted by the University College London Committee for the Ethics of non–National Health Service Human Research, and all aspects of data collection and storage were in accordance with the standards stipulated by this body. Informed consent for both study participation and publication were obtained from all participants.

### Measurement of emotional over- and under-eating

Emotional over- and under-eating were measured using the Child Eating Behaviour Questionnaire (CEBQ), a parent report questionnaire consisting of 35 items capturing eight distinct eating behaviours hypothesized to play a causal role in childhood weight variation. The EOE (4 items; e.g.: “My child eats more when annoyed”) and EUE (4 items; e.g.: “My child eats less when upset”) scales were used in the present study^17^. Items were rated along a 5-point Likert scale (‘never’; ‘rarely’; ‘sometimes’; ‘often’; ‘always’) and mean scores were calculated for participants whose parents had completed a minimum of 3 out of 4 items for each subscale. EOE and EUE scores were available for a total of 2054 children, which included 346 MZ twin pairs and 681 DZ twin pairs. The CEBQ is the most widely used psychometric measure of children’s eating behaviour with good internal reliability (EOE: α = 0.72–79, EUE: α = 0.74–0.75), good test-retest reliability over a two week period^17^, and has been validated against laboratory-based measures of eating behaviour^21^. Both scales had good internal reliability in this sample (EOE: α = 0.71, EUE: α = 0.81).

### Zygosity, age and gestational age

Parents reported the weight at birth of each twin, taken from the child’s personal health record, as well as gestational age. The exact age of the twins at data collection was calculated from the twins’ date of birth and the date the questionnaire was completed. At baseline, opposite sex twin pairs were classified as DZs. For same sex pairs, parents were asked to complete the 18-item Zygosity Questionnaire for Young Twins. This questionnaire was specifically developed to aid zygosity classification in young twin pairs and has been validated longitudinally and against DNA markers^22^. DNA from a random sample of 81 twin pairs was used to validate the zygosity questionnaire in Gemini. DNA confirmed 43 pairs as MZ and 38 as DZ; which exactly matched the results of the questionnaires (Herle *et al*., 2016).

### Statistical analyses

Pearson’s correlation coefficient was used to examine the association between EOE and EUE prior to twin analyses. In line with common practice EOE and EUE scores were regressed on sex, gestational age and age at measurement, to ensure that factors shared completely by twin pairs did not contribute to similarity between pairs and inflate the shared environmental effect. Further to remove any positive skew, EOE and EUE scores were log transformed prior to analyses.

#### Twin analyses

The twin method exploits the natural occurrence of identical and non-identical twin pairs. Identical twins [MZ] share 100% of their genetic material, whereas non-identical [DZ] twins share approximately 50% of their segregating genes. However, both types of twins share their environments to a very similar extent (e.g. gestated in the same mother, born at the same time, raised in the same family). This means MZ and DZ twins can be compared, and greater similarity among MZ than DZ pairs indicates a genetic contribution to variation in a trait because the only real difference between the two types of twins is that MZ pairs are twice as similar genetically. Comparing MZ and DZ correlations (or covariation) enables variation in the trait to be decomposed into three latent factors: additive genetic effects (A); shared environmental influence (C); and non-shared environmental influence (E). The shared environmental influence comprises environmental factors that contribute to similarity between twins in a pair, such as socio-economic status; non-shared environmental influences contribute to differences within twin pairs, such as twins attending different schools or illness experienced by only one twin. The bigger the difference in resemblance between MZs and DZs, the greater the genetic contribution to variation in the trait; the greater the similarity between twin pairs regardless of zygosity the more important the contribution from the shared environment; and any differences between MZ twins (who share both their genetic and shared environmental factors completely) quantifies the extent of unique environmental influence (as well as measurement error)^23^.

This concept can be extended to understand the extent of *common* genetic and environmental influences underlying two different traits – i.e. shared aetiology. The basis of the bivariate method is to compare the correlation (or covariation) between two different phenotypes, across twin pairs (so-called a cross-twin cross-trait correlation, CT-CT); resemblance in CT-CTs for MZ and DZ pairs is then compared, using the same principles as the univariate method. A higher CT-CT correlation for MZ pairs relative to DZ pairs indicates that genetic factors contribute to the phenotypic association between the two traits; similar CT-CT correlations for both types of twins indicates that shared environmental effects are important in driving the phenotypic association; no CT-CT correlation indicates that unique environmental influences are driving the phenotypic association between the two traits. The bivariate method therefore decomposes *covariation* between two traits into A, C and E; while the univariate method decomposes *variation* in a single trait into A, C and E^23^. Genetic and environmental contributions to variation and covariation in EOE and EUE were estimated using intraclass correlations (ICCs) and Maximum Likelihood Structural Equation Modelling (MLSEM).

#### Intraclass correlation analyses

MZ and DZ intraclass correlations (ICCs) were calculated for EOE and EUE scores to examine patterns of resemblance for EUE and EOE. CT-CT correlations were calculated for MZ and DZ twins to observe patterns of resemblance for covariation between EOE and EUE. ICCs and CT-CT correlations were calculated using OpenMx in R^24^.

#### Maximum likelihood structural equation modelling

Maximum Likelihood Structural Equation Modelling (MLSEM) was used to provide reliable parameter estimates of A, C and E with 95% confidence intervals and goodness-of-fit statistics. First a saturated model was computed with no parameter constraints; i.e. it estimates all possible means, variances and covariances for both EOE and EUE, for MZs and DZs. The specified ACE model was then compared to this model for goodness-of-fit.

A bivariate Correlated Factors Model was used to estimate A, C and E underlying the variation in EOE and EUE, and the covariation between them. The Correlated Factors Model produces etiological correlations (additive genetic correlation [r_A_], shared environmental correlation [r_C_], and non-shared environmental correlation [r_E_]) that indicate the extent to which the A, C and E influences underlying the two behaviours are the same or different. The etiological correlations can be interpreted along similar lines to a Pearson’s correlation coefficient. For example, a high *positive* r_A_ indicates that many of the same genetic factors that predispose to EOE also predispose to EUE, whereas a low r_A_ suggests that different genetic factors influence the two behaviours. In addition, a high *negative* r_A_ would indicate that many of the same genetic factors that make a child *more likely* to engage in EOE would also make a child *less likely* to engage in EUE.

*Bivariate* estimates of A, C and E are also produced; these indicate the proportion of the *phenotypic association* between EOE and EUE explained by common genetic (bivariate A), common shared environmental (bivariate C) or common unique environmental (bivariate E) influences underlying the two behaviours. The bivariate estimates are calculated by dividing the covariance of the latent factors (A, C and E) by the phenotypic correlation between the two variables. If all bivariate estimates are in the same direction, negative or positive, they can be expressed as a proportion of the phenotypic correlation between the two variables. Importantly, etiological correlations and bivariate estimates are independent of one another. It is therefore possible for the majority of the genetic influences underlying EOE and EUE to be the same, but for common shared environmental influences to play the most important role in driving the phenotypic association between them (e.g. a high r_A_, and a high bivariate C)^25^.

MLSEM was carried out using OpenMx software^24^. The Likelihood Ratio Test (LRT) was chosen as an appropriate model fit indicator. The LRT compares the fit value (−2 Log likelihood) of the specified ACE model, to the fit value of the saturated model (in which it is nested). The p-value indicates how well the specified ACE model fits, compared to a fully saturated model. A significant p-value (<0.05) indicates that the ACE model has a significantly *worse* fit to the data; a non-significant difference in model fit (p > 0.05) indicates that the ACE model provides an acceptable fit to the data^26^.

#### Sensitivity analyses

Prior to analyses full sex limitation models were conducted to test for the presence of sex differences between boys and girls. Fit statistics indicated some sex differences for EOE, however none of the parameter estimates were significantly different for boys and girls. There were no sex differences in the aetiology underlying EUE. A model combining males and females was therefore used. Estimates and fit statistics for the sex limitation models can be found in the supplementary information (Supplementary Information, Tables 1 to 4).

The subscales of the CEBQ are correlated with one another: food approach behaviours are positively correlated (Food Responsiveness (FR), Enjoyment of Food (EF), Desire to Drink (DD) and EOE), as are food avoidant behaviours (Food Fussiness (FF), Satiety Responsiveness (SR), Slowness of Eating (SE) and EUE); and food approach behaviours are negatively correlated with food avoidance behaviours. In order to establish the specific aetiology of EOE and EUE (independently of the other CEBQ eating behaviours), sensitivity analyses were conducted on EOE scores that had been regressed on all of the other food approach behaviours (FR, EF and DD) and on EUE that had been regressed on all of the other food avoidant behaviours (FF, SR and SE). There were no differences between the estimates derived from the original analyses and the sensitivity analyses. We therefore present only the original analyses in the manuscript, and provide the sensitivity analyses in the supplementary information (Supplementary Information, Table 5).

## Results

Characteristics of the sample are shown in Table 1. EOE and EUE were significantly positively correlated (r = 0.43, p < 0.001), indicating that children who emotionally overeat tend also to emotionally under-eat.

**Table 1:** Descriptive Statistics for sample included in these analyses.

Twin pairs	N (%) or Mean (SD)
**Total**	1027 pairs (2054 children)
**Zygosity**	
MZ^1^ pairs	346 (34)
DZ^1^ pairs	681 (66)
**Sex of pairs**	
MZ Male-Male	176 (17.2)
DZ Male-Male	165 (16.1)
MZ Female-Female	169 (16.5)
DZ Female-Female	199 (19.4)
DZ Opposite Sex	316 (30.8)
**Gestational age (weeks)**	36.26 (2.43)
**Weight at birth (kg)**	2.46 (0.54)
**Weight at birth SDS** ^1^	−0.58 (0.92)
**BMI** ^1^ **SDS at 5 years**	−0.22 (1.10)
**Age at 5 year questionnaire (years)** ^1^	5.15 (0.13)
**Emotional Overeating at 5 years**	1.56 (0.51)
**Emotional Undereating at 5 years**	2.66 (0.84)

^1^MZ: Monozygotic; DZ: Di-zygotic; SDS: Standard Deviation Score; BMI: Body Mass Index Age at 5 year questionnaire: Exact age when questionnaire was filled in was calculated using the date of birth of the twins and the date when the questionnaire was filled in.

### Intraclass correlations (ICCs)

ICCs for EOE and EUE were calculated for MZ and DZ twin pairs separately to examine the patterns of resemblance for each behaviour. As shown in Table 2, the ICCs were high and of similar magnitude for both MZs and DZs, for both EUE and EOE. This pattern of twin correlations suggests strong shared environmental factors underlying variation in both EUE and EOE. The cross-twin cross-trait (CT-CT) correlations showed a similar pattern to the univariate ICCs (also shown in Table 3). The CT-CT correlations for both MZ and DZ pairs were significant, of similar magnitude, and of a comparable effect size to the phenotypic correlation itself indicating that shared environmental influences are largely driving the observed phenotypic association between EOE and EUE.

**Table 2:** Intraclass correlations (ICCs) and cross-twin cross-trait (CT-CT) correlations for EOE and EUE measured at 5 years.

	**MZ** ^1^	**DZ** ^1^
EOE ICCs^1^ (95% CI^1^)	0.98 (0.97–0.98)	0.94 (0.92–0.94)
EUE ICCs (95% CI)	0.98 (0.97–0.98)	0.95 (0.94–0.95)
CT-CT (95% CI)	0.43 (0.42–0.43)	0.44 (0.39–0.49)

^1^MZ: Monozygotic; DZ: Di-zygotic; EOE: Emotional Overeating; ICCs: Intraclass correlations; CI: Confidence intervals; CT-CT: Cross-twin cross-trait.

**Table 3:** Model fit statistics for saturated and bivariate model.

Model	parameters	−2LL^1^	df^1^	Δ χ² (df)	p-value	AIC^1^
Sat^1^	28	−1542.604	4078			−9698.604
ACE^1^	11	−1518.404	4095	24.199 (17)	0.11	−9708.404

^1^Abbreviations: 2LL: −2 times log-likelihood of data; df: degrees of freedom; Δ χ²: change in chi-square; AIC: Akaike’s Information Criterion; Sat: Saturated model; ACE: Full bivariate Correlation Factors Model.

### Maximum Likelihood Structural Equation Modelling (MLSEM)

The bivariate ACE Correlated Factors model (including all parameters; A, C, and E for EOE and EUE; r_A_, r_C_, and r_E_ between EOE and EUE) was tested against the saturated model. The LRT indicated no significant difference in fit between the two models (Δ χ² = 24.199, p = 0.11), confirming that the bivariate ACE Correlated Factors Model fitted the data well. In line with the LRT, Aikaike’s Information Criterion (AIC) favored the bivariate Correlated Factors Model over the saturated model, indicated by the lower value. Fit statistics for the saturated and bivariate Correlated Factors Model are shown in Table 3.

Parameter estimates for A, C and E (and 95% confidence intervals, CIs) indicated the relative importance of genetic, shared environmental and unique environmental influences on variation in EOE and EUE. As suggested by the ICCs, variation in both EOE and EUE was largely explained by shared environmental influences (EOE: C = 0.90, 95% CI: 0.89, 0.92; EUE: C = 0.91, 95% CI: 0.90, 0.92). In contrast, genetic effects only played a minor role in explaining variation in either of the two behaviours (EOE: A = 0.07, 95% CI: 0.06, 0.09; EUE: A = 0.07, 95% CI: 0.06, 0.09). Contributions from non-shared environmental factors were also small (EOE: E = 0.02, 95% CI: 0.02, 0.03; EUE: E = 0.02, 95% CI: 0.02, 0.02).

**Figure 1:**
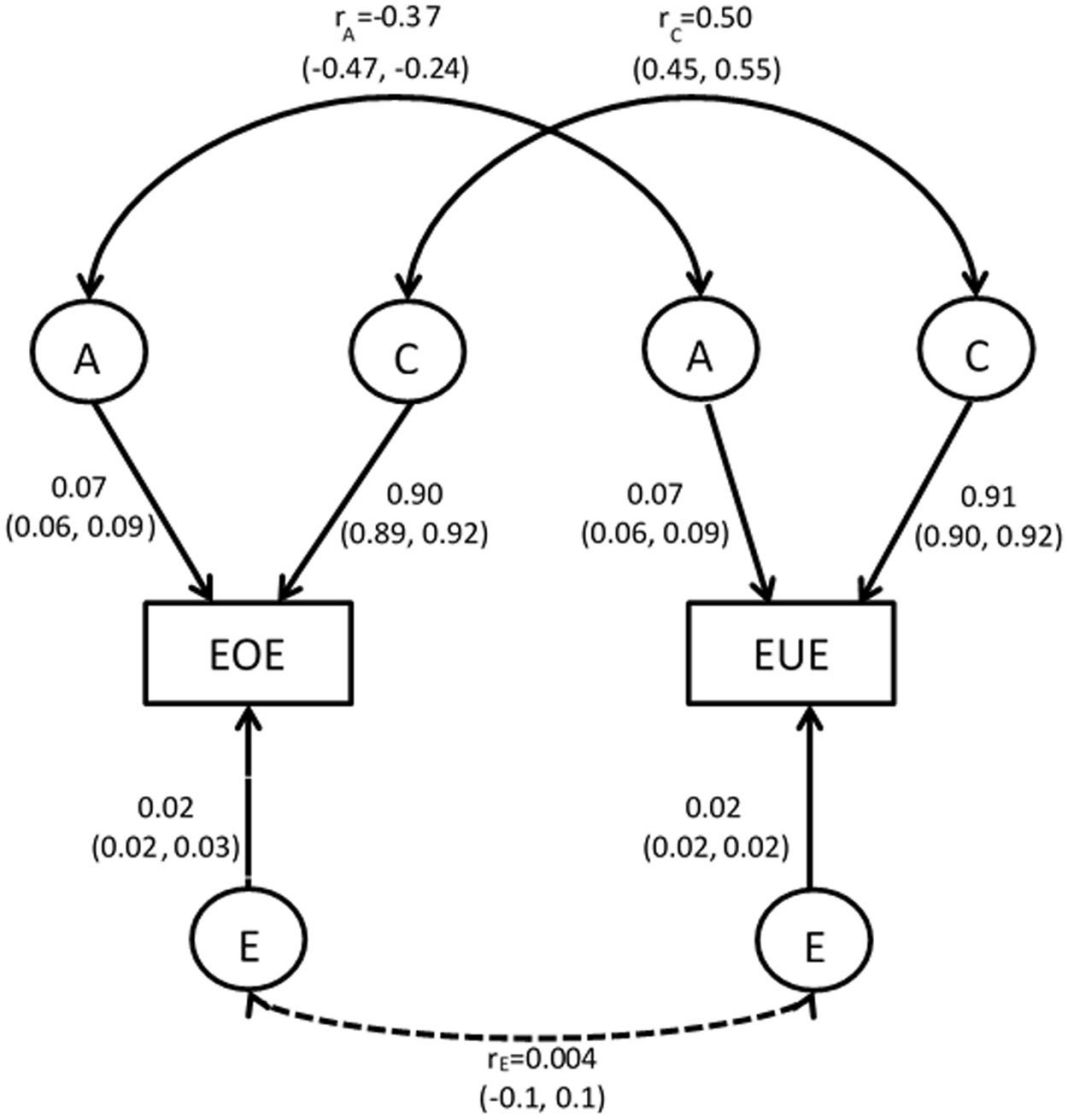
Full bivariate Correlated Factors Model, including all parameters. The rectangular boxes represent the measured phenotype (emotional overeating, EOE and emotional under-eating, EUE) using the Child Eating Behaviour Questionnaire at five years of age. The circles indicate the latent factors: additive genetic effects (A), shared environmental effects (C) and non-shared environmental effects (E). The straight single-headed arrows reflect pathways with the variance explained by each latent factor (including 95% confidence intervals, CI). The etiological correlations are shown on the curved double-headed arrows. These indicate the extent of common genetic (r_A_), shared environmental (r_C_) and non-shared environmental (r_E_) influences across the two phenotypes. The non-significant etiological correlation (r_E_), with a 95% CI crossing 0, is represented as a dotted line. Bivariate estimates (not shown on the path diagram) quantify the proportion of the phenotypic association (r = 0.41, p < 0.001) attributable to genetic (bivariate A = −0.03, 95% CI: −0.004, −0.02), shared environmental (bivariate C = 0.44, 95% CI: 0.39, 0.48) and unique environmental factors (BivE: 0.00, 95% CI: −0.00, 0.00) that are common to both EOE and EUE.

The etiological correlations are displayed in Fig. 1. The shared environmental correlation (r_C_) was significant, positive and moderate in effect size (r_C_ = 0.50, 95% CI: 0.44, 0.55) indicating that a quarter (0.50^2^) of the shared environmental influences that predispose a child to engage in EOE are the same as those that predispose a child to engage in EUE. There was also a significant negative genetic correlation which was moderate in effect size (r_A_ = −0.38, 95% CI: −0.51, −0.26). However, because the genetic contributions to variation in EUE and EOE were so small (7%), the genetic correlation between them is difficult to interpret. The correlation for non-shared environmental effects was non-significant (r_E_ = 0.02, 95% CI: −0.1, 0.1).

The bivariate estimates indicated that common shared environmental factors underlying both EOE and EUE were largely driving the observed phenotypic association between them (BivC = 0.45, 95% CI: 0.40, 0.50). In contrast bivariate A was very small (BivA = −0.03, 95% CI: −0.04, −0.02); and bivariate E was estimated as zero (BivE = 0.00, 95% CI: −0.00, 0.00).

## Discussion

### Summary of findings

This is the first twin study to establish the relative importance of genetic and environmental influences on variation in EUE, and the extent to which emotional *under-* and *overeating* share their aetiology. In line with our previous study of EOE^19^, we found individual differences in EUE are largely explained by shared environmental factors (91%) in early childhood, while genetic influences play only a minor role (7%). The very low heritability estimates for both of these behaviours were somewhat surprising, and in stark contrast to the much higher heritability estimates observed for a range of other eating behaviours in both infants and children, captured using the CEBQ and the Baby Eating Behaviour Questionnaire, the infant version which includes four subscales: food responsiveness, satiety responsiveness, slowness in eating and enjoyment of food^27, 28^. In infancy, heritability is moderate to high for four eating behaviours (53–84%)^27^; in toddlers, heritability is high for ‘food fussiness’ (78%)^29^, and in 10-year-old children heritability is high for both ‘satiety responsiveness’ (63%) and ‘food responsiveness’ (75%)^30^.

EOE and EUE were moderately positively correlated, in line with previous studies^9, 17, 18^ indicating that children who tend to emotionally overeat tend also to emotionally under-eat. Common shared environmental influences largely explained the observed association between these two behaviours; in other words, the reason EOE and EUE are positively correlated is because there are a number of shared environmental factors that shape the development of *both* of these behaviours. Children’s eating behaviour is influenced by a multitude of factors - individual and environmental^31^ - and parenting behaviours have received most attention^32^. Although the present inquiry did not examine which environmental factors are at play, longitudinal research has shown that parents who use food to soothe their child when upset (so-called ‘emotional feeding’) encourage the development of emotional overeating in their school-aged children^33^; cross-sectional studies have also reported associations between emotional feeding and EOE^34, 35^. Cross-sectional studies have also reported associations between other parental feeding practices and higher EOE in two to five year-old children, including exerting high levels of pressure on a child to eat, and being overly restrictive about *what* or *how much* the child is allowed to eat^14, 36^. A recent study showed that five to seven year old children consumed more calories from snack foods when exposed to a mild stressor. Children were more likely to consume greater amounts when experiencing negative emotions if their parents reported using food as a reward and greater restriction of food for health reasons two years earlier^37^. The present findings indicate that many of the factors that shape EOE are the same as those that shape EUE. It is therefore reasonable to assume that parents’ feeding practices may affect EUE as well, although research is needed to confirm this assumption. For example, parents who exert high levels of pressure on their children to eat might induce stress during mealtimes, making it more likely that the child will become anxious around food, especially when upset. Parental pressure to eat is associated with lower food intake^38, 39^; EUE might be one of the behaviours that explains this.

Research has suggested that a lack of social support and a more negative family environment are associated with EUE, e.g. children whose parents have a more hostile relationship with each other were found to engage more in EUE^40^. Further evidence comes from a retrospective study of adult women diagnosed with anorexia nervosa, who reported both a lack of social support and having engaged in EUE during their childhood^41^. It is possible that lack of social support may be particularly important for the development of EUE but further research is needed.

To our knowledge no previous theory has been developed to explain why some individuals are more likely to **both** over- **and** under-eat in response to stress. Observational studies have shown that individuals differ substantially in their response to emotional stress, insofar as some increase their food intake, while others decrease it^1^. But this research does not address the issue of the same individuals responding differently on different occasions. Early animal research, however, suggested that stress intensity might contribute to response differences. The appetite and grooming behaviors of rats have been shown to differ in response to low and chronic^42^ or intense stress (electric shock)^43^. It may therefore be the case that the same individual may be prone to undereating in response to a certain type of stress (e.g. intense acute stress), but tends to overeat in response to low-level chronic stress (e.g. certain aspects of the family environment). However, even when exposed to the same stressors, rats showed large variation in appetite response, pointing towards the contribution of individual factors^44^. A child’s ability to regulate their emotion may be one of the common factors that explains the positive association between EOE and EUE. It might be the case that children who are less able to manage their own emotions through positive strategies, have a tendency for their eating behaviour to be affected under emotions of stress; and the intensity and type of stressor determines whether the child ends up over- or under-eating. Child emotion regulation has in fact been shown to predict a parents’ tendency to use food to sooth, and to predict the tendency of a child to engage in EOE; i.e. parents are more likely to emotionally feed their child if he or she has greater negative affectivity, and emotional feeding contributes to increasing EOE as time goes on (in a reciprocal, progressive manner)^45^. Less is known about the shapers of EUE, but parental feeding strategies may also play a role. A potential theoretical model of EOE and EUE may therefore include stress intensity, emotional regulation ability and parental feeding strategies. Future research needs to examine the expression of EOE and EUE in the context of different stressors of varying intensity, children’s ability to self-regulate their emotions, and parental feeding strategies. Novel theoretical models also need to account for EOE and EUE having opposite effects on weight and weight gain, in spite of their positive association.

This is the first twin study of EUE, so it is not possible to compare the findings with any other study. However, there have been three adult twin studies of *EOE*, although none of which observed any shared environmental effects, with all the variance in EOE being explained by genetic and *non-shared* environmental effects. Overall, these adult studies have also reported larger genetic influences on EOE than were found in the present study, although estimates varied widely (9%–60%) due to limited sample sizes and wide age ranges^46–48^. Outcomes from twin studies are age and sample specific and findings from adult studies cannot be extrapolated to children. This is of particular importance for twin studies because genetic influence tends to increase steadily with development for a variety of phenotypes (e.g: IQ^49^ and BMI^50^); something that may indicate active gene-environment correlation^51^ – seeking out the opportunity to act upon genetic predispositions, which increases as children mature and gain autonomy. It would be interesting to establish the heritability of EUE in an adult sample to ascertain if genetic influence is higher. The recent development of the Adult Eating Behaviour Questionnaire^52^, which includes both EOE and EUE scales, makes this possible.

This research has increased our understanding of the aetiology of EOE and EUE. Our findings suggest that emotional eating is a learned behaviour in early childhood, and that many of the underlying environmental factors are the same for EOE and EUE. This suggests that the same factors can be addressed to prevent both EOE and EUE in children. Preventive efforts are required as emotional eating increases the risk for developing over- and underweight in childhood^9, 11^ and is associated with later symptoms of eating disorders^53–55^.

### Limitations

EOE and EUE were parent reported, and the measures could be subject to bias. Direct observations and laboratory tests would be advantageous, and have been used to validate some of the CEBQ subscales^21^. However such methods are time consuming and costly for large samples and therefore pose practical issues for large scale research. Nevertheless, it would be useful to apply the methods developed by a few other researchers who have been able to measure EOE objectively in a laboratory setting in order to validate the CEBQ measures of EOE and EUE. Additionally, the EOE and EUE subscales tap different emotions: EOE relates to annoyance, worry, anxiety and boredom; EUE to feeling upset, tired, angry, and unhappy. It is possible that parental feeding practices differ depending on the emotion in question, such as feeding to sooth in response to a child’s sadness but not in response to anger. Future research needs to investigate more thoroughly the complex relationships between parental feeding and child emotional eating, acknowledging the different negative emotional states. It is possible that the aetiology of eating in response to anger differs from the aetiology of overeating when feeling sad. Future research would therefore benefit from differentiating emotional over and under-eating by different emotional states. Parents are deemed to know their children better than anyone else and are arguably the best informants of their children’s eating behaviour. In addition, EOE and EUE scores may be influenced to some extent by the parents’ own tendencies to emotionally eat, or by their emotional feeding practices. Both might have resulted in parents scoring the two children more similarly, inflating the shared environmental effect (because both twins would be rated similarly regardless of their zygosity). However, this bias should apply equally to other parent-reported child eating behaviours (e.g. food fussiness), yet EOE and EUE show much higher shared environmental influence and much lower genetic influence in comparison^27–29^; suggesting that parents can and do distinguish between their child and their own behaviour. Lastly, this is the first twin study of EUE, replication is therefore necessary.

## Conclusion

EOE and EUE in early childhood are learned, not inherited. The most important environmental shapers of these behaviours are those that are entirely shared by twin pairs, such as parental and family factors. The reason EOE and EUE are *positively* correlated is because many of the shared environmental influences that shape them are the same. Future research should aim to establish which environmental factors are associated with both EOE and EUE in childhood. High influence of the shared environment suggests emotional eating is modifiable in early life.

## Electronic supplementary material

Supplementary information
