# Supplementary material for: Emotional over- and under-eating in early childhood are learned not inherited
Source: Sci Rep. 2017 Aug 22;7:9092. doi: 10.1038/s41598-017-09519-0 (PMC5567210; doi:10.1038/s41598-017-09519-0)
Supplement: Supplementary file 1 — Supplementary information [file 41598_2017_9519_MOESM1_ESM.doc]

**Title: Emotional over- and under-eating in early childhood are learned not inherited**

Authors: Moritz Herle, Dr Alison Fildes, Dr Silje Steinsbekk, Frühling Rijsdijk and Dr Clare H Llewellyn

**Supplementary information**

**Sex limitation models**

**Supplemental Table 1** Parameters estimates (95% Confidence intervals) for A, C and E for males and females considering qualitative and quantitative sex differences in EOE age 5 years

| **Model** | **Male** | | | **Female** | | |  |  |
| --- | --- | --- | --- | --- | --- | --- | --- | --- |
|  | **A_m_^1^** | **C_m_^1^** | **E_m_^1^** | **A_f_^1^** | **C_f_^1^** | **E_f_^1^** | **r_A_^1^** | **r_C_^1^** |
| **Full sex limitation (r_A_=free)** | 0.07 (0.05, 0.10) | 0.90 (0.88, 0.92) | 0.02 (0.02, 0.03) | 0.10 (0.07, 0.12) | 0.87 (0.85, 0.90) | 0.03 (0.02, 0.04) | 0.5 (0.47, 0.5) | 1.00 |
| **Full sex limitation**  **(r_C_=free)** | 0.07 (0.05, 0.10) | 0.90 (0.88, 0.92) | 0.02 (0.02, 0.03) | 0.10 (0.07, 0.12) | 0.87 (0.85, 0.90) | 0.03 (0.02, 0.04) | 0.5 | 1.00  (0.99-1.00) |
| **Common effects model** | 0.07 (0.05, 0.10) | 0.90 (0.88, 0.92) | 0.02 (0.02, 0.03) | 0.10 (0.07, 0.12) | 0.87 (0.85, 0.90) | 0.03 (0.02, 0.04) | 0.5 | 1.00 |
|  | **A** | | **C** | | **E** | | **Scalar** | |
| **Scalar Model** | 0.07  (0.06, 0.09) | | 0.90  (0.89, 0.92) | | 0.02  (0.02,0.03) | | 1.01  (0.98, 1.05) | |
|  | **A** | | **C** | | **E** | | **r_A_** | **r_C_** |
| **Null model (no sex differences)** | 0.09  (0.07, 0.11) | | 0.89  (0.87, 0.90) | | 0.02  (0.02, 0.03) | | 0.5 | 1.00 |

^1^ Abbreviations: A_m_: additive genetic component of variance for males; C_m_: shared environmental component of variance for males; E_m_: unique environmental component of variance for males; A_f_: additive genetic component of variance for females; C_f_: shared environmental component of variance for females; E_f_: unique environmental component of variance for females; r_A_: genetic correlation, r_c_ : shared environmental correlation, r_E_ : non-shared environmental correlation.

**Supplemental Table 2** Fit statistics for sex limitation models for EOE at age 5 years

| **Model** | **Comparison** | **Ep^1^** | **-2LL^1^** | **Df^1^** | **Δ χ² (df)^1^** | ***p* value** | **AIC^1^** |
| --- | --- | --- | --- | --- | --- | --- | --- |
| 1 Saturated model |  | 23 | -1740.449 | 2029 |  |  | -5798.449 |
| 2 Full sex limitation (r_A_=free) | 1 | 9 | -1689.445 | 2043 | 51.004 (14) | <0.001 | -5775.445 |
| 3 Full sex limitation (r_C_=free) | 1 | 9 | -1689.445 | 2043 | 51.004 (14) | <0.001 | -5775.445 |
| 4 Common effects model (r_A_=0.5, r_C_=1) | 2 & 3 | 8 | -1689.445 | 2044 | 0.00 (1) | 1 | -5777.445 |
| 5 Scalar Model | 4 | 6 | -1684.942 | 2046 | 4.503 (2) | 0.11 | -5776.942 |
| 6 Null model (no sex differences) | 4 | 5 | -1684.418 | 2047 | 5.027 (3) | 0.17 | -5778.418 |

Abbreviations: Ep: estimated parameters, 2LL: -2 times log-likelihood of data; df: degrees of freedom; Δ χ² 2: change in chi-square; AIC: Akaike’s Information Criterion; Sat: Saturated model; r_A_: genetic correlation, r_c_ : shared environmental correlation

**Supplemental Table 3** Parameters estimates (95% Confidence intervals) for A, C and E for males and females considering qualitative and quantitative sex differences in EUE at 5 years

| **Model** | **Male** | | | **Female** | | |  |  |
| --- | --- | --- | --- | --- | --- | --- | --- | --- |
|  | **A_m_^1^** | **C_m_^1^** | **E_m_^1^** | **A_f_^1^** | **C_f_^1^** | **E_f_^1^** | **r_A_^1^** | **r_C_^1^** |
| **Full sex limitation (r_A_=free)** | 0.07 (0.05, 0.09) | 0.91 (0.89, 0.94) | 0.02 (0.02, 0.03) | 0.07 (0.05, 0.09) | 0.91 (0.89, 0.94) | 0.02 (0.02, 0.03) | 0.36 (0.11, 0.5) | 1.00 |
| **Full sex limitation**  **(r_C_=free)** | 0.07 (0.03, 0.09) | 0.91 (0.89, 0.94) | 0.02 (0.02, 0.03) | 0.07 (0.05, 0.09) | 0.91 (0.89, 0.94) | 0.02 (0.01, 0.02) | 0.5 | 0.99 (0.98, 1.00) |
| **Common effects model**  **(rA=0.5, r_C_=1)** | 0.08 (0.05, 0.10) | 0.90 (0.88, 0.93) | 0.02 (0.02, 0.03) | 0.07 (0.04, 0.09) | 0.91 (0.88, 0.93) | 0.02 (0.01, 0.02) | 0.5 | 1.00 |
|  | **A** | | **C** | | **E** | | **Scalar** | |
| **Scalar Model** | 0.07  (0.06, 0.09) | | 0.91  (0.89, 0.92) | | 0.02  (0.02, 0.02) | | 1.01  (0.98, 1.04) | |
|  | **A** | | **C** | | **E** | | **r_A_** | **r_C_** |
| **Null model (no sex differences)** | 0.07  (0.06, 0.09) | | 0.91  (0.89, 0.92) | | 0.02  (0.02, 0.02) | | 0.5 | 1.00 |

^1^ Abbreviations: A_m_: additive genetic component of variance for males; C_m_: shared environmental component of variance for males; E_m_: unique environmental component of variance for males; A_f_: additive genetic component of variance for females; C_f_: shared environmental component of variance for females; E_f_: unique environmental component of variance for females; r_A_: genetic correlation, r_c_ : shared environmental correlation, r_E_ : non-shared environmental correlation.

**Supplemental Table 4** Fit statistic for sex limitations models for EUE at 5 years

| **Model** | **Comparison** | | **Ep^1^** | **-2LL^1^** | **Df^1^** | **Δ χ² (df)^1^** | ***p* value** | **AIC^1^** |
| --- | --- | --- | --- | --- | --- | --- | --- | --- |
| 1 Saturated model | |  | 23 | 399.1595 | 2031 |  |  | -3662.84 |
| 2 Full sex limitation (r_A_=free) | | 1 | 9 | 418.8832 | 2045 | 19.724 (14) | 0.139 | -3671.117 |
| 3 Full sex limitation (r_C_=free) | | 1 | 9 | 418.8832 | 2045 | 19.724 (14) | 0.139 | -3671.117 |
| 4 Common effects model (r_A_=0.5, r_C_=1) | | 2 & 3 | 8 | 420.1546 | 2046 | 1.271(1) | 0.260 | -3671.845 |
| 5 Scalar Model | | 4 | 6 | 421.8833 | 2048 | 1.729 (2) | 0.421 | -3674.117 |
| 6 Null model (no sex differences) | | 4 | 5 | 422.0701 | 2049 | 1.915 (3) | 0.590 | -3675.93 |

Abbreviations: Ep: estimated parameters, 2LL: -2 times log-likelihood of data; df: degrees of freedom; Δ χ² 2: change in chi-square; AIC: Akaike’s Information Criterion; Sat: Saturated model; r_A_: genetic correlation, r_c_ : shared environmental correlation

**Supplemental Table 5** Parameter estimates (95%CI) for A, C and E as well as aetiological correlations for EOE and EUE at five years; first as presented in the manuscript and then with scores regressed by other CEBQ eating behaviours

| **Findings presented in manuscript** | | | | | | | | |
| --- | --- | --- | --- | --- | --- | --- | --- | --- |
| **EOE** | | | **EUE** | | | **Aetiological correlations** | | |
| **A** | **C** | **E** | **A** | **C** | **E** | **r_A_** | **r_C_** | **r_E_** |
| 0.07  (0.06, 0.09) | 0.90  (0.89, 0.92) | 0.02  (0.02, 0.03) | 0.07  (0.06, 0.09) | 0.91  (0.90, 0.92) | 0.02  (0.02, 0.02) | -0.37  (-0.47,-0.24) | 0.50  (0.45, 0.55 | 0.004  (-0.1, 0.1) |
| **Sensitivity analyse, including EOE and EUE regressed for other food approaching/avoidant eating behaviour** | | | | | | | | |
| **EOE** | | | **EUE** | | | **Aetiological correlations** | | |
| **A** | **C** | **E** | **A** | **C** | **E** | **r_A_** | **r_C_** | **r_E_** |
| 0.11  (0.09, 0.14) | 0.85  (0.82, 0.87) | 0.04  (0.03, 0.5) | 0.13  (0.10, 0.15) | 0.84  (0.82, 0.87) | 0.03  (0.03, 0.04) | -0.33  (-0.47,-0.19) | 0.44  (0.38, 0.50) | 0.13  (0.02, 0.23) |

Abbreviations: A: additive genetic component of variance; C: shared environmental component of variance; E: unique environmental component of variance; r_A_: genetic correlation, r_c_ : shared environmental correlation, r_E_ : non-shared environmental correlation.
